# Targeting the Immune System with Plant Lectins to Combat Microbial Infections

**DOI:** 10.3389/fphar.2017.00671

**Published:** 2017-10-04

**Authors:** Jannyson J. B. Jandú, Roberval N. Moraes Neto, Adrielle Zagmignan, Eduardo M. de Sousa, Maria C. A. Brelaz-de-Castro, Maria T. dos Santos Correia, Luís C. N. da Silva

**Affiliations:** ^1^Departamento de Bioquímica, Centro de Biociências, Universidade Federal de Pernambuco, Recife, Brazil; ^2^Pós-Graduação em Biologia Parasitária, Universidade Ceuma, São Luís, Brazil; ^3^Núcleo de Enfermagem, Universidade Federal de Pernambuco, Vitória de Santo Antão, Brazil

**Keywords:** immunomodulatory lectins, host–parasite interaction, immunization, adjuvants, new treatments

## Abstract

The arsenal of drugs available to treat infections caused by eukaryotic and prokaryotic microbes has been declining exponentially due to antimicrobial resistance phenomenon, leading to an urgent need to develop new therapeutic strategies. Host-directed immunotherapy has been reported as an attractive option to treat microbial infections. It consists in the improvement of host defenses by increasing the expression of inflammatory mediators and/or controlling of inflammation-induced tissue injury. Although the *in vitro* antimicrobial and immunomodulatory activities of lectins have been extensively demonstrated, few studies have evaluated their *in vivo* effects on experimental models of infections. This review aims to highlight the experimental use of immunomodulatory plant lectins to improve the host immune response against microbial infections. Lectins have been used *in vivo* both prophylactically and therapeutically resulting in the increased survival of mice under microbial challenge. Other studies successfully demonstrated that lectins could be used in combination with parasite antigens in order to induce a more efficient immunization. Therefore, these plant lectins represent new candidates for management of microbial infections. Furthermore, immunotherapeutic studies have improved our knowledge about the mechanisms involved in host–pathogen interactions, and may also help in the discovery of new drug targets.

## Introduction

Through centuries, microbial infectious diseases continue to be among the leading causes of mortality and morbidity worldwide (Morens and Fauci, 2013; Sands et al., 2016; Rogalski et al., 2017). No doubt, the antibiotics discovery in the 1930s has revolutionized medicine and changed the treatment of infectious diseases, resulting in a dramatic increase in life expectancy and quality (Aminov, 2016). However, ever since these drugs were introduced, microbial resistance has evolved and spread very rapidly (Davies and Davies, 2010). Indeed, eukaryotic and prokaryotic microbes can acquire drug resistance by several mechanisms [for review see (Van Acker et al., 2014; Blair et al., 2015; Fairlamb et al., 2016; Goncalves et al., 2016; Hall and Mah, 2017)]. This fact critically reduces the shelf life of antibiotics that are not efficient to combat the emerging multidrug-resistant strains (Holmes et al., 2016; Laxminarayan et al., 2016). In addition, pathogens have developed several mechanisms to evade and suppress the host defenses and/or to induce exacerbated inflammation (which may cause host tissue injury) (Chaves et al., 2016; Gomes et al., 2016; Ko, 2016; Malachowski et al., 2016; Sha et al., 2017). This scenario encourages the development of new approaches to treat microbial infection, such as those based on the modulation of the host immune system. The immunomodulatory therapies are based in the stimulation of specialized and specific host immune responses against microbes rather than target microbe viability or virulence (Hancock et al., 2012; Czaplewski et al., 2016; Fura et al., 2017).

Among the natural products, plant lectins are known as potent immunomodulatory agents, able to act in both innate and adaptive immune system. They modulate the production of cytokines and other mediators of immune response (such as reactive oxygen and nitrogen species), and, thus, improve the defenses against microbes (Souza et al., 2013; da Silva and Correia, 2014; Coelho et al., 2017). Plant lectins comprise one heterogeneous class of proteins with at least one non-catalytic carbohydrate-binding domain (Coelho et al., 2017). The lectin–carbohydrate interactions have been associated with several biotechnology applications (Komath et al., 2006; de Oliveira Figueiroa et al., 2017). In several cases, the immunomodulatory activity of plant lectins was associated with their interaction with glycan moieties present on the surface of immune cells. Such interaction can result in signal transduction which triggers the effector mechanisms involved in the response against microbial infections (Souza et al., 2013).

Plant lectins play a crucial role in the protection against microbial phytopathogens (Hwang and Hwang, 2011; Kim et al., 2015). Based on this, the antimicrobial and antivirulence actions of several lectins have been demonstrated *in vitro* against different bacteria of medical importance, as reviewed by several authors (Islam and Khan, 2012; Dias Rde et al., 2015; Coelho et al., 2017; Palharini et al., 2017). Other works have demonstrated the antibacterial effects of lectins using *in vitro* cell-based assays. For example, a lectin isolated from *Aegle marmelos* fruit inhibited the adherence and invasion of *Shigella dysenteriae* to human colonic epithelial cells (HT29 cells), protecting these cells against cell death (induced through apoptosis) (Raja et al., 2011). Similarly, the chitin-binding lectin isolated from the juicy sarcotesta of *Punica granatum* (named PgTel) showed to have broad-spectrum antibacterial action (inhibiting Gram-positive and Gram-negative bacteria). PgTel was also able to inhibit the invasion of some bacteria to HeLa cells (human epithelioid cervix carcinoma) (Silva et al., 2016b).

The immunomodulatory effects of plant lectins on different immune cells have been also addressed by several authors (Unitt and Hornigold, 2011; Pereira-da-Silva et al., 2012; Souza et al., 2013; da Silva and Correia, 2014; Coelho et al., 2017). Based on this, some works have demonstrated that some well-known immunomodulatory lectins are able to enhance the phagocytic ability of immune cells and their cytokine production in the presence of bacteria (da Silva et al., 2015b; Batista et al., 2017). This review aims to highlight the use of immunomodulatory plant lectins in contending infection provoked by bacterial, fungal, and protozoan pathogens. The lectins selected for this review did not exhibit direct inhibition of microbial growth (using *in vitro* assays), thus their *in vivo* actions are related to their ability to target the immune system. Initially, these *in vivo* studies were primarily focused on mouse models of infection and are summarized in **Table 1**.

**Table 1:** Application of immunomodulatory lectins in *in vivo* experimental models of microbial infections.

Lectin	Plant species	Sugar specificity	Pathogen	Type of use	Reference
Artin M	*Artocarpus integrifolia*	Mannose	*Candida albicans*	Prophylactic	Custodio et al., 2011
			*Paracoccidioides brasiliensis*	Therapeutic	Coltri et al., 2008
			*Leishmania* spp.	Adjuvant	Panunto-Castelo et al., 2001; Teixeira et al., 2006
			*Toxoplasma gondii*	Therapeutic	Santana et al., 2014
			*Neospora caninum*	Adjuvant	Cardoso et al., 2011
CFL	*Cratylia argentea*	Glucose/mannose	*Salmonella enterica*	Prophylactic	Silva et al., 2016a
ConA	*Canavalia ensiformis*	Mannose	*Klebsiella pneumoniae*	Prophylactic and therapeutic	Kuo et al., 2007
			*Candida albicans*	Prophylactic	Loyola et al., 2002; Moresco et al., 2002
ConBr	*Canavalia brasiliensis*	Glucose/mannose	*Salmonella enterica*	Prophylactic	Silva et al., 2016a
Cramoll	*Cratylia mollis*	Mannose	*Cryptococcus gattii*	Therapeutic	Jandú et al., 2017
Jacalin	*Artocarpus integrifolia*	Galactose	*Trypanosoma cruzi*	Adjuvant	Albuquerque et al., 1999
			*Neospora caninum*	Adjuvant	Cardoso et al., 2011
ScLL	*Synadenium carinatum*	Galactose	*Leishmania amazonensis*	Prophylactic	Afonso-Cardoso et al., 2011
			*Neospora caninum*	Adjuvant	Cardoso et al., 2012
			*Toxoplasma gondii*	Therapeutic	Santana et al., 2014


## Plant Lectins to Combat Bacterial Infections

**FIGURE 1:**
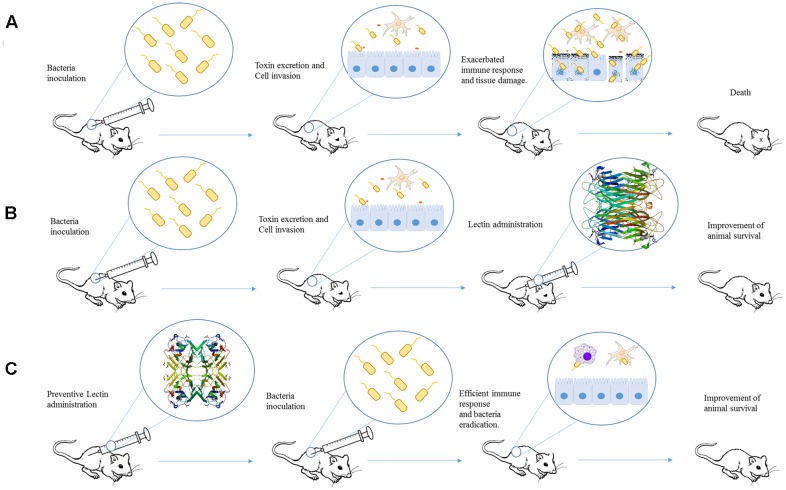
Schematic representation of studies employing plant lectins in experimental bacterial infection. **(A)** After inoculation, bacteria can invade cell and provoke damage by releasing different virulence factors and inducing an exacerbated immune response. The final effect is organ dysfunction and animal death. **(B)** Administration of a lectin (for example, ConA) after infection could improve mice response against bacterial infection and increase the animal survival. **(C)** Pre-treatment of animal with lectin (such as ConBr) induces an immune response able to protect against bacterial virulence resulting in the improvement of animal survival. The proteins structures were obtained from Protein Data Bank, the ID codes are 4PF5 and 4P14 for ConA and ConBr, respectively.

As mentioned before several papers have investigated the potential of plant lectins direct inhibit bacterial growth (Souza et al., 2013; da Silva and Correia, 2014; Coelho et al., 2017), however, only few experimental studies are available about their *in vivo* effects (as illustrated in **Figure 1**). These lectins are well known due to their ability to modulate the host immune system).

### Benefits of Prophylactic and Therapeutic Treatments with Concanavalin A in *Klebsiella pneumoniae* Infection

The most studied plant lectin is Concanavalin A (ConA) which is isolated from *Canavalia ensiformis*. ConA is able to stimulate the proliferation of immune cells and enhance the expression of toll-like receptors (Sodhi et al., 2007; da Silva and Correia, 2014). In order to evaluate whether ConA immunomodulatory properties could inhibit a bacterial infection, a murine model based on intragastrical inoculation of *Klebsiella pneumoniae* was employed (Kuo et al., 2007). *K. pneumoniae* is a pathogen commonly associated with nosocomial infections that can invade tissues provoking damage on essential organs (such as liver necrosis) and sepsis (Wu et al., 2017). Although ConA had no effect on the *in vitro* bacterial growth, beneficial effects were observed when infected mice were submitted either to prophylactic or therapeutic treatment with this lectin. The pretreatment (2 h before infection) with single doses of ConA (1 mg/kg or 2 mg/kg) enhanced the mice survival to 55% after 9 days of infection (the survival rate for untreated infected mice was 10%). When ConA (2 mg/kg) was administered in consecutives doses (2 h before infection followed by other doses at 48 and 96 h), the animal survival rate was 83% (the best effect observed in this study). The last treatment was based on the administration of two successive doses at 24 or 72 h after infection, resulting in a 50% of mice survival for ConA at 2 mg/mL. The effects of ConA were further demonstrated by the inhibition of liver necrosis induced by *K. pneumoniae*, reduced levels of aspartate aminotransferase and alanine aminotransferase, and bacterial survival in blood and liver (Kuo et al., 2007).

### ConBr and CFL Have Prophylactic Effects on *Salmonella enterica* Infection

Recently, the prophylactic effects of the lectins isolated from *Canavalia brasiliensis* (ConBr) and *Cratylia argentea* (CFL) were evaluated in an experimental model of *Salmonella enterica* serovar Typhimurium infection (Silva et al., 2016a). Both lectins were previously reported as immunomodulatory agents (de Oliveira Silva et al., 2011). Firstly, the authors showed that these lectins did not have anti-*S. enterica* activity in concentrations ranging from 0.019 to 10,000 μg/mL. Afterward, each lectin was inoculated into Swiss mice (intraperitoneal route; i.p.) at different concentrations (1, 5, and 10 mg/kg) 1 day prior bacterial infection (i.p.). Both lectins showed a 70–80% dose-dependent survival rate increase effect after 7 days of treatment, for ConBr and CFL, respectively. When the lectins were administered (at 10 mg/kg) daily for 3 days prior to bacterial infection (i.p. route), the survival ratios were 90% for CFL and 100% for ConBr. The authors also showed reduction of bacterial growth into the peritoneal cavity, bloodstreams, spleen, and the liver of lectins pre-treated animals. Furthermore, both lectins reduced the amounts of TNF-α and IL-10 cytokines in the peritoneal fluid, but IL-1 was only reduced using ConBr (Silva et al., 2016a). In a later paper, these lectins were shown to inhibit the colonization of Swiss mice peritoneal macrophage by *Salmonella*, through modulation of the expression of TLR and inflammatory mediators (cytokines and nitric oxide) (Batista et al., 2017).

## Plant Lectins for Treatment of Fungal Infections

Despite the use of antifungal agents, the invasive fungal infections are responsible for high rates of morbidity and mortality (Badiee and Hashemizadeh, 2014; Camplesi et al., 2017; Enoch et al., 2017). For example, cryptococcosis is responsible for 1 million cases of meningoencephalitis, especially in HIV positive individuals, with 624,000 death per year (McMullan et al., 2013). Yeasts from *Candida* genus, particularly *Candida albicans*, are commonly found as etiological agent of neonatal bloodstream infections (Fu et al., 2017; Vaezi et al., 2017).

Other example is the neglected disease paracoccidioidomycosis, the most important systemic mycosis in Latin America (mainly in Brazil) with high mortality rates (de Macedo et al., 2016). In fact, paracoccidioidomycosis is the eighth most important cause of mortality among chronic infectious diseases, reaching rates of 1.65 deaths per 106 inhabitants (de Oliveira et al., 2015). Collectively, these invasive fungal infections are more prevalent in immunocompromised patients (Woyciechowsky et al., 2011; Kaur et al., 2017; Sungkanuparph et al., 2017). In addition, they are in general also associated with antifungal resistance, making their treatment ineffective for most cases (Gullo et al., 2013; Chowdhary et al., 2014; Scorzoni et al., 2017).

Taken together, these factors point out the need of studies, using both *in vitro* and *in vivo* models, for the development of new therapeutic alternatives to treat fungal infections. In this scenario, lectins with immunomodulatory compounds have been emerging as promising options (Armstrong-James and Harrison, 2012; Datta and Hamad, 2015). Cytokines, antibodies, opsonins, and immunomodulatory compounds (combined or not with antifungals drugs) are therapeutic alternatives for the treatment of fungal infections (Armstrong-James and Harrison, 2012; Datta and Hamad, 2015; Posch et al., 2017; Scorzoni et al., 2017), such as cryptococcosis (Antachopoulos and Walsh, 2012), invasive *Candida* infections (Safdar et al., 2004), and aspergillosis (Stuehler et al., 2011). In fungal infection models, large number of lectins have been applied *in vitro* and *in vivo* in order to develop new antifungal strategies (Islam and Khan, 2012; Coelho et al., 2017). Some examples of plant lectins able to modulate fungal infections are provided below.

### ConA Pretreatment Protects Mice from *Candida albicans* Infection

The effects of ConA in an experimental model of *C. albicans* infection have been associated with activation of antifungal responses by increasing of phagocytosis and killing of yeast cells by macrophages and neutrophils (Loyola et al., 2002; Moresco et al., 2002). However, the literature does not describe any direct effects of ConA on *C. albicans* viability or virulence. In the first paper, by Loyola et al. (2002), ConA was intraperitoneally administrated and, after 6 h, the collection of neutrophils and macrophages from peritoneal exudate was performed. ConA administration increased the number of peritoneal cells and their *in vitro* ability to kill *C. albicans* (in both yeast and germ tube forms) and increased the expression of mannose receptors. Furthermore, ConA pre-treatment also increased the survival of animals challenged with *C. albicans* (6 h after the lectin inoculation) (Loyola et al., 2002). These data were confirmed by a similar work where ConA efficiently promoted the antifungal action of peritoneal macrophages from suckling and adult mice by increasing the phagocytosis and killing of *C. albicans*. This paper also showed that ConA protected suckling mice against intraperitoneal infection with *C. albicans* (Moresco et al., 2002).

### Artin M Has Prophylactic and Therapeutic Effects on Fungal Infections

The mannose-specific lectin present in *Artocarpus integrifolia* (Moraceae) seeds, nominated Artin M, is a well-known immunomodulatory protein able to stimulate neutrophils migration by haptotaxis (Ganiko et al., 2005; Souza et al., 2013). This capacity is due to its interaction with mannose residues, commonly found at extracellular matrix components (such as laminin), helping the cell migration into injured tissues. This is an important phenomenon in the inflammatory response against infections (Ganiko et al., 2005; Souza et al., 2013). *A. integrifolia* seeds are also sources of jacalin, a galactose-binding lectin with the characteristic beta-prism-I fold (Raval et al., 2004). This domain consists in four-stranded beta-sheets and the lectins with this domain are assembled in a family called Jacalin-related lectins (JRL) (Esch and Schaffrath, 2017).

The prophylactic inoculation (3 days before infection) of the crude extract of *A. integrifolia* seeds (containing Artin M and jacalin) resulted in the enhanced survival and reduced liver injury of Swiss mice infected with *C. albicans*. These effects were not observed when mice were treated with jacalin alone. Using the same protocol, the authors showed that the Artin M alone or in combination with jacalin induced a Th1 and Th17 response mediated by dectin-1 and mannose receptors, resulting in a significant increase of TNF-α production, phagocytic and candidacidal activities (Custodio et al., 2011). Similarly, it was demonstrated that Artin M increased the TNF-α production and phagocytic activity of *C. albicans* by mice macrophages. These actions of Artin M were mediated by dectin-1 and mannose receptors (Loyola et al., 2012).

In addition, Artin M (in both native and recombinant form) also showed efficacy against the infection caused by *Paracoccidioides brasiliensis* (Coltri et al., 2008). The authors performed an elegant work where they first determined that the best treatment schedule consisted in the subcutaneous administration of Artin M in single dose (0.5 μg of KM in 50 μL of PBS) and 10 days after infection with *P. brasiliensis*. Mice treated with Artin M displayed reduced levels of yeasts on their lungs and consequently less pulmonary lesions. These effects were induced through production of IL-12 by a TLR-2 dependent mechanism (Coltri et al., 2008).

### Cramoll Has Therapeutic Benefits in Mice Infected with *Cryptococcus gattii*

A recent paper reported the use of the lectin purified from seeds of *Cratylia mollis* (pCramoll or Cramoll 1,4) for the treatment of mice infected with *Cryptococcus gattii*. *C. mollis* is an endemic plant of Caatinga (Brazil semi-arid area), a plant from the Brazilian exclusive biome. pCramoll is a mannose-specific lectin and it has shown several biotechnological applications, including induction of cell proliferation (Maciel et al., 2004; da Silva et al., 2015a), *in vitro* immunomodulation (de Melo et al., 2010; da Silva et al., 2015b), wound healing (Albuquerque et al., 2017), and anticancer properties (da Cunha et al., 2016). Particularly, the immunomodulatory ability of pCramoll has been demonstrated in an *in vivo* model of wound healing in immunocompromised mice (de Oliveira Silva et al., 2011) and in experimental infection with *Schistosoma mansoni* (de Oliveira Silva et al., 2011). Cramoll, however, did not show antimicrobial activity *in vitro*.

**FIGURE 2:**
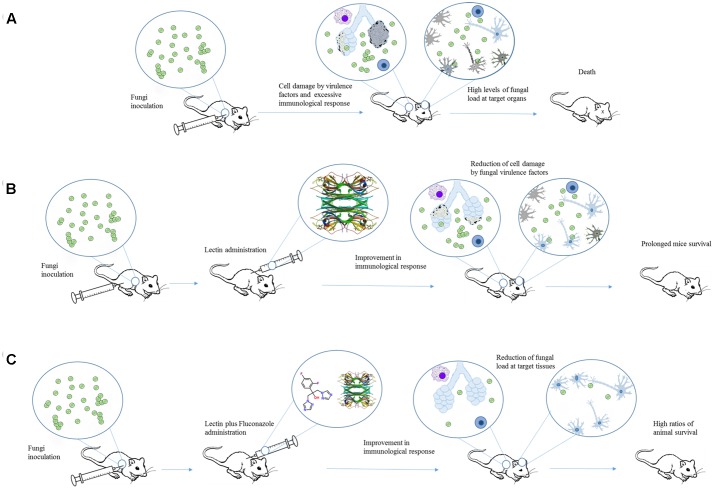
An overview of the effects of Cramoll alone or in combination with fluconazole in an experimental cryptococcosis model. **(A)** *Cryptococcus gattii* provokes tissue damage and organ dysfunction by releasing different virulence factors and inducing an exacerbated immune response. **(B)** When Cramoll was administrated alone **(B)** or in combination with fluconazole **(C)**, infected mice exhibited increased ratios of survival and reduced levels of morbidity and behavior alteration. The PDB code for Cramoll structure is 1MVQ.

Initially, pCramoll was administered in different concentrations (1, 250, and 500 μg) 1 day before the intratracheal infection with *C. gattii*. Afterward, every 10 days after infection a new dose of lectin was given to the mice. pCramoll enhanced the mice survival equally in all tested concentrations. When combined with fluconazole (azole antifungal drug), the best results were found for this lectin at 1 μg. pCramoll alone or in combination with fluconazole decreased pulmonary fungal burden of mice. These effects were associated with an increase of inflammatory infiltrate on the lungs, and modulatory action on cytokines levels (down-regulation of IFNγ, IL-6, and IL-10 and up-regulation of IL-17A). The combined treatment of pCramoll and fluconazole also significantly decrease the fungal load in the brain, reducing the morbidity and behavior changes caused by the infection (i.e., neuropsychiatric state, motor behavior, autonomic function, tone and muscle strength, and reflex/sensory function). Moreover, *in vitro* analysis revealed that bone marrow-derived macrophages treated with pCramoll were more able to phagocyte *C. gattii*, with higher production of reactive oxygen species, and decreased the intracellular fungal proliferation (Jandú et al., 2017). These findings are summarized in **Figure 2**.

## Plant Lectins and Protozoan Infections

Some plant lectins (Jacalin, Artin M, and ScLL) have been also successfully applied against infections caused by protozoan. These lectins were used in infections caused by *Trypanosoma cruzi* (Albuquerque et al., 1999), *Leishmania* spp. (Panunto-Castelo et al., 2001), *Neospora caninum* (Cardoso et al., 2011, 2012), and *Toxoplasma gondii* (de Souza et al., 2016). In these studies, the lectins were used also as adjuvants in combination to parasite antigens in order to induce a more efficient immunization.

### Jacalin as Adjuvant in *Trypanosoma cruzi* Infection

**FIGURE 3:**
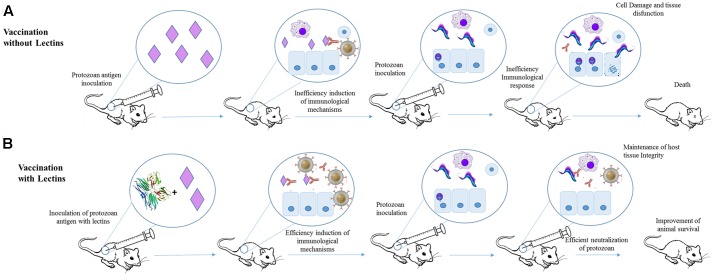
Application of Jacalin as adjuvant for immunization against *T. cruzi*. **(A)** Mice were immunized with *T. cruzi* antigens and after 1 month infected with *T. cruzi*. The antigens failed in inducing an efficient protective humoral response. **(B)** Animal subjected to immunization with jacalin (PDB ID 1JAC) plus *T. cruzi* antigens showed higher antibodies titers and lower parasitemia levels than mice that received only *T. cruzi* antigens.

The effects of jacalin in the humoral immune response toward *T. cruzi* infection were evaluated using Balb/c mice (Albuquerque et al., 1999). Jacalin is able to modulate cellular and humoral immunity, which makes it a potential candidate for use as an adjuvant compound (Miyamoto et al., 2012; Danella Polli et al., 2016). Initially, the animals were inoculated with *T. cruzi* antigens in the presence or not of Jacalin. The mice submitted to immunization with *T. cruzi* antigens plus Jacalin produced more antibodies (and faster) than animals immunized only with parasite antigens. The efficiency of immunization using *T. cruzi* antigens plus Jacalin was also demonstrated by challenging 1-month-old immunized mice with trypomastigotes. These animals showed reduced levels of parasitemia when compared to non-immunized mice. Similarly, mice immunized with viable *T. cruzi* epimastigotes (at 1.0 × 10^5^ or 1.0 × 10^6^) plus Jacalin produced more antibodies than mice inoculated with parasites alone. However, the combined inoculation of 1.0 × 10^5^ *T. cruzi* epimastigotes plus jacalin resulted in lower levels of parasite after 9 days of infection than animals immunized only with epimastigotes. Jacalin alone did not protected the animals from infection (Albuquerque et al., 1999; **Figure 3**).

### Artin M Is a Potent Adjuvant in Leishmaniasis Model

**FIGURE 4:**
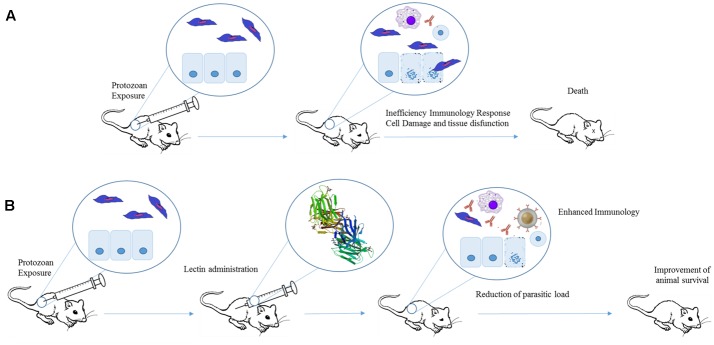
Therapeutic effects of plant lectins in protozoan infections. Experimental exposure of mice to protozoan results in cell damage, tissue destruction, and consequently animal death **(A)**. Lectins can improve protozoan-infected animal survival by increasing production of Th1 cytokines. The induction of pro-inflammatory response leads to a reduction in parasite levels and organ dysfunction **(B)**. The structure of Artin M was obtained from PDB (ID: 1J4U).

The evidence that Artin M could be useful to treat protozoan infections was obtained from the ability of this lectin to induce the expression of IL-12p40, which could drive the production of Th1 cytokines instead of the Th2 pattern, typical of unresponsive parasite infections (**Figure 4**). The authors performed a combined administration of Artin M and soluble leishmanial antigen (SLA) into the footpad of BALB/c mice. SLA injected animals showed higher levels of IL-4 than the group treated with SLA+Artin M, while the IFN-γ concentration was higher in SLA+Artin M group. The animals treated with Artin M alone or SLA+Artin M were also more resistant to *Leishmania major* infection, and these mice showed smaller lesions than those groups treated with SLA alone or untreated animals (Panunto-Castelo et al., 2001).

Recently, it was shown that Artin M improved the *in vitro* killing of *L. major* by neutrophils through modulation of effector mechanisms, such as enhanced excretion of inflammatory cytokines, reactive oxygen species, and neutrophil elastase and myeloperoxidase. In addition, the infected-neutrophils treated with Artin M did not form neutrophil extracellular traps and showed shorter life span than untreated infected cells, both characteristics that may favor the maintenance of host tissue integrity (Ricci-Azevedo et al., 2016).

Artin M was also effective as an adjuvant of SLA in immunization against *Leishmania amazonensis* (Teixeira et al., 2006). Artin M+SLA administration reduced the parasite amounts in the footpad of mice infected with *L. amazonensis* 15 days after immunization, although the lesion size was not reduced. Mice treated only with Artin M showed smaller lesion and decreased parasite load in relation to the untreated group (but the levels of parasites were not smaller than Artin M+SLA group). Other pro-inflammatory plant lectins (ConBr and PAA purified from *Pisum arvense*) were not able to inhibit the lesion size in mice infected with *L. amazonensis*, even when inoculated in combination with SLA. However, the association of ConBr and SLA resulted in smaller number of parasites in the footpad of immunized animals when compared to the controls (Teixeira et al., 2006).

### ScLL and Prophylactic Treatment of *L. amazonensis*

The lectin obtained from leaves latex of *Synadenium carinatum* (ScLL) has also shown protective effects in a murine model of leishmaniasis induced by *L. amazonensis*. In this study, BALB/c mice received three doses of ScLL (10, 50, or 100 μg/animal) in the presence or not of SLA (25 μg/animal) with intervals of 15 days. Three days after this immunization, the animals were infected with *L. amazonensis* promastigotes in their left footpad. When administrated alone at 100 μg/animal, ScLL were more effective than when associated with SLA (as shown by reduction of lesion size and parasite load). Thus, this lectin showed a better potential as prophylactic agent than as adjuvant. The mice treated with SLA also showed higher levels of IgG2a and Th1 cytokine expression (IFN-γ, IL-12, and TNF-α) (Afonso-Cardoso et al., 2007). *In vitro* cell-based analysis showed that ScLL reduced the association of macrophages and *L. amazonensis*, inducing the production of pro-inflammatory cytokines (IL-1, IL-12, and TNF-α) in a nitric oxide independent pathway (Afonso-Cardoso et al., 2011).

### Adjuvant Properties of Artin M, Jacalin, and ScLL in Experimental Neosporosis

*Neospora caninum* (Apicomplexa), etiologic agent of neosporosis, is a prevalent intracellular parasite associated with cases of abortion in cattle and neuromuscular disease in dogs (Donahoe et al., 2015). The potential adjuvant actions of Artin M, Jacalin, and ScLL have been evaluated in murine models of neosporosis. In the first report, C57BL/6 mice received (subcutaneously) three doses with 2-week intervals of *N. caninum* lysate antigen (NLA; 25 μg/animal) associated with Artin M (1 μg/animal) or Jacalin (100 μg/animal). Animals immunized with Artin M+NLA showed higher levels of specific antibodies against *N. caninum* (IgG, IgG1, and IgG2a) than all others groups in all times evaluated (15, 30, and 45 days after immunization). The association of Jacalin and NLA also enhanced the levels of total IgG in relation to animals immunized with NLA alone in all times, however, the levels of IgG1 were only higher until 30 days after immunization. Animals immunized with Jacalin and NLA showed similar levels of IgG2 than NLA group. The mice were then infected with lethal doses of *N. caninum* tachyzoites 60 days after immunization. Artin M+NLA combination resulted in 86% of protection, while the other immunized groups (NLA+JAC, NLA, Artin M, or JAC) were partially protected. In addition, Artin M+NLA reduced the number of parasites in the brain and induced a more robust inflammatory profile. The results highlight that Artin M has more potential to be used as adjuvant for neosporosis than Jacalin (Cardoso et al., 2011).

Cardoso et al. (2012) demonstrated the adjuvant and immunomodulatory effects of ScLL in a similar work. The authors showed that C57BL/6 mice dendritic cells produced inflammatory cytokines when treated with NLA+ScLL or ScLL alone. NLA (25 μg/animal) associated with ScLL (1 μg/animal) were inoculated in C57BL/6 mice three time for 45 days. The animals that received NLA + ScLL produced higher levels of IgG and IgG1 than the NLA immunized mice. The NLA+ScLL and ScLL groups were also more resistant to infection by *N. caninum* tachyzoites (which occurred 60 days after the last immunization) (Cardoso et al., 2012).

### Artin M and ScLL for Therapy of Acute Toxoplasmosis

Recently, the therapeutic properties of both Artin M and ScLL were studied in a model of murine toxoplasmosis. For this, C57BL/6 mice were orally infected with cysts of *T. gondii*, and treated intraperitoneally for 6 days with ScLL (50 μg), Artin M (1 μg), or ScLL (50 μg) plus Artin M (1 μg). The treatment with ScLL was more efficient, resulting in 100% survival, while 60% of the Artin M + ScLL-treated animals and only 40% of Artin M-treated group survived. The best results obtained with ScLL alone are related to its capacity to induce the production of Th1 cytokines (IL-2, IFN-γ, and IL-6) resulting in reduced levels of parasite in the brain (de Souza et al., 2016). Other study showed the action of eutirucallin on the *in vitro* infection of human foreskin fibroblasts (HFF) by *T. gondii* (Palharini et al., 2017). Eutirucallin is a RIP-2 type lectin obtained from the latex of *Euphorbia tirucalli*, which also displays immunostimulatory action (increasing neutrophils migration and release of NO, IL12p40, and TNF-α by peritoneal macrophages) (Santana et al., 2014). Eutirucallin inhibited infection and intracellular replication of *T. gondii* with IC50 of 173.2 and 133.3 μg/mL, respectively (Palharini et al., 2017).

## Conclusion and Perspectives

The high immunomodulatory abilities of plant lectins were proven efficient to combat microbial infections in different experimental models. Depending on the type of lectin and the kind of infection, the lectin showed better prophylactic or therapeutic behavior. As inductors of Th1 response, some lectins were used as adjuvant agents. These immunotherapeutic studies have also improved our knowledge about the pathogen–immunity relationship, and could be helpful to provide insights for the development of new therapeutic strategies. All these successful examples of plant lectins encourage the study of others lectins with immunomodulatory capabilities for the treatment of infectious diseases. However, it is always important to remind that the possible adverse effects (for example, TNF-α-mediated hepatitis, renal and intestinal injury) should be evaluated before the clinical application of these plant lectins. The side effects depend on type of lectin, dose, and administration route. To the best of our knowledge, the anti-infective effects of plant lectins have not been clinically evaluated in humans (although some lectins have been tested in clinical trials for cancer therapy).

Another challenge is the purification yields that for some lectins could not be suitable for large-scale production. The advances in protein engineering (recombinant protein production, structure–function improvement) and drug delivery technologies (liposomes, microcapsules, etc.) may improve the protein production, stability, and their pharmacokinetics properties (delivery, bioavailability, controlled release, and targetability). These actions may result in enhanced therapeutic index and may reduce likely side effects. Furthermore, the combination of *in silico* approaches and analytical tools for protein have provided more insight in lectin and ligand interactions. In summary, these stimulating research data pave the way for the future use of plant lectin as immunomodulatory agents to combat microbial infections.

## Author Contributions

JJ, RMN, AZ, EdS, MB, MdSC, and LdS: Contributed to conception, design, and critically revised the manuscript. All authors gave final approval and agree to be accountable for all aspects of the work.

## Conflict of Interest Statement

The authors declare that the research was conducted in the absence of any commercial or financial relationships that could be construed as a potential conflict of interest.
